# Recombinant bovine somatotropin in the synchronization of ovulation in crossbred dairy cows (*Bos taurus indicus × Bos taurus taurus*)

**DOI:** 10.14202/vetworld.2020.746-750

**Published:** 2020-04-21

**Authors:** Fabrício Albani Oliveira, Ítalo Câmara de Almeida, Larissa Marchiori Sena, Jurandy Mauro Penitente-Filho, Ciro Alexandre Alves Torres

**Affiliations:** 1 Federal Institute of Espirito Santo, Alegre, Espirito Santo, 29520-000, Brazil; 2 Department of Veterinary Medicine, Federal University of Espirito Santo, Alegre, Espirito Santo, 29500-000, Brazil; 3 Pitágoras College, Ipatinga, Minas Gerais, 35160-294, Brazil; 4 Department of Animal Science, Federal University of Viçosa, Campus Universitário, Viçosa, Minas Gerais, 36571-000, Brazil

**Keywords:** biotechnology, dairy cows, fertility, follicle, reproduction

## Abstract

**Aim:**

The aim of this study was to evaluate the effects of the administration of recombinant bovine somatotropin (rbST) at the moment of implementation of the timed artificial insemination protocol, on follicular dynamics and pregnancy rate in crossbred cows.

**Materials and Methods:**

A total of 346 cows were used in two experiments with a factorial 2×2 design. The cycling cows (Tcycling) and the anestrous cows (Tanestrous) were considered as factor 1 and the administration of rbST (TrbST) or not (Tcontrol) as factor 2. The experimental protocol: (1) Tcontrol – day 0 (D0), insertion of a progesterone-release intravaginal device (PRID) plus 2 mg of estradiol benzoate (EB); D8, PRID removal, plus 0.150 mg of prostaglandin F_2α_, and 400 IU of equine chorionic gonadotropin; D9, 1 mg of EB; and with artificial insemination at day 10; (2) TrbST – similar to Tcontrol plus 500 mg of rbST on D0. In experiment I, ultrasound examinations were performed in all treatments. In experiment II, the cows’ pregnancy rate was evaluated. Data were analyzed with 5% probability.

**Results:**

There was no effect of the protocols on cows cyclicity or follicular growth rate (p>0.05). There was no interaction of the effects, administration of rbST, and the cyclicity of cows on the pregnancy rate. The total pregnancy rate observed was 49.0%. The pregnancy rate in cows receiving rbST was lower for anestrous compared with cycling cows (p<0.05).

**Conclusion:**

The administration of rbST did not alter the patterns of follicular dynamics nor the ovulation rate. However, cows in anestrous that received rbST had lower pregnancy rates than cycling cows.

## Introduction

Timed artificial insemination (TAI), determined by the synchronization of ovulation, has increased in Brazil as a consequence of the increased use of the technology by producers. At present, results of TAI are similar to those observed with artificial insemination (AI) [1]. Recombinant bovine somatotropin (rbST) hormone is used in dairy cows to increase milk production. However, its effects on their reproduction require further investigation [2]. It has been shown that rbST stimulates the production of endogenous growth hormone and insulin-like growth factor I (IGF-I), which are crucial for reproduction. In addition, the presence of rbST receptors in the uterus and ovaries has previously been observed [3].

Changes caused by the use of rbST are probably due to its combined direct effect and the indirect effect of IGF-I. It was observed that the use of rbST increases the number of small follicles in the ovaries [4,5]. The IGFs are important in follicular growth by stimulating the proliferation of granulosa cells synergistically with the gonadotropins [6,7]. Receptors for rbST and IGF-I are also located in the endometrial glands [8]. It has been suggested that an increase of IGF-I, as a consequence of the use of rbST, enhances the secretion activity of endometrium glands, leading to an improvement of the uterine environment for the conceptus, and development and maintenance of pregnancy [9]. The use of rbST may also increase interferon-tau secretion by the conceptus [10].

In this context, the objective of this study was to evaluate the effects of rbST use on TAI protocols in crossbred dairy cows (*Bos taurus taurus* × *Bos taurus indicus*) on the follicular dynamics and reproductive efficiency.

## Materials and Methods

### Ethical approval

This study was in accordance with the Ethical Principles of Animal Experimentation, adopted by the Ethics Committee on the Use of Animals (CEUA-UFES), under number 017/2014.

### Location

The experiment was carried out between May 2015 and October 2015, on a commercial farm located in the municipality of Dores do Rio Preto, State of Espirito Santo, Brazil, at the geographic coordinates 20° 41’ South, 41° 50’ West, and 774 m altitude. The farm is located in a region with hot/rainy summers and cold/dry winters, with an average annual temperature of 19.2°C and annual rainfall of 1000 mm.

### Animals and feeding

A total of 346 crossbred milking cows (*B. t. indicus* × *B. t. taurus*) within the first and fourth lactation were selected based on their fertility background and lack of clinical signs of infectious or metabolic diseases, retention of placenta, or abnormal genitals during the gynecologic examination. Cows were kept on pastures of *Brachiaria brizantha* cv. Marandu and water and mineral salt were administered *ad libitum* and supplemented with concentrate containing 22% of crude protein at 1 kg/3 L of milk.

### Experimental treatments and variables analyzed

During the application of the progesterone-release intravaginal device (PRID), the period postpartum was recorded relative to the beginning of the TAI protocol (day 0). Milk production and cows’ body weight (BW) were obtained and the body condition score (BCS, ranging from 1 to 5) was also measured [11]. Cows were uniformly divided into groups based on their parturition order, milk production, and BCS. Cows had an average BW of 408±1.6 kg, average BCS of 2.4±0.0, average parturition of 2.3±0.2, and average milk production of 11.6±1.0 L.

Two experiments were performed in a 2***×***2 simultaneous factorial design (cyclicity of the cows and use of rbST). Follicular dynamics was evaluated in experiment I, while the reproductive efficiency of the cows was evaluated in experiment II. The cows cyclicity corresponded to cycling (Tcycling) and anestrous (Tanestrous) cows, and the use (TrbST) or not (Tcontrol) of rbST. The physiological status of the cows was determined by the presence (cycling cows) or absence of a corpus luteum (anestrous cows) by ultrasound and rectal palpation 7 days before the beginning of the ovulation synchronizing protocol.

In experiment I, cycling cows (n=20) and anestrous cows (n=20) were assigned into two groups: Group 1: Tcontrol (n=20 cows) – day 0 (D0), insertion of PRID (Cronipress^®^, Biogénesis Bagó, Argentina) plus intramuscular injection of 2 mg of estradiol benzoate (EB) (Bioestrogen^®^, Biogénesis Bagó, Argentina); day 8 (D8), the PRID was removed and 0.150 mg of prostaglandin F_2α_ (PGF_2α_; Croniben^®^, Biogénesis Bagó, Argentina) and 400 UI of equine chorionic gonadotropin (eCG; Novormon 5000^®^, Syntex S.A., Argentina) were injected intramuscularly; day 9 (D9), intramuscular injection of 1 mg of EB; and day 10 (D10), cows were artificially inseminated 52 h after the removal of the PRID. Group 2: TrbST (n=20 cows) – similar to Tcontrol, where on day 0, 500 mg of rbST (Boostin 500^®^, Coopers Brasil) was injected. Therefore, a total of 10 cows were assigned in each treatment: Tcycling/Tcontrol, Tanestrous/Tcontrol, Tcycling/TrbST, and Tanestrous/TrbST, respectively.

Ultrasound evaluations were performed on days 0-8 and on the day of TAI using a portable ultrasound coupled with a 5.0 MHz linear rectal probe (MINDRAY^®^, model DP2200 VET). On the day of TAI, the follicle dynamic was also evaluated at with intervals of 12 h until ovulation was determined by lack of the dominant follicle. The follicle status was thus evaluated on D0, while the follicle dynamicity and ovulation index were evaluated on the remaining days. Follicles were classified according to their diameter as small (SF <6 mm), medium (MF 6-8 mm), and dominant (DF >8 mm) [12]. Growth index (mm/day) was then calculated by the difference between the diameter of the follicles on days 10 and 8, divided by the number of days.

In experiment II, cows were uniformly assigned by parturition, milk production, BW, and BCS as proposed in the experiment I: Tcycling/Tcontrol (n=86), Tanestrous/Tcontrol (n=78); Tcycling/TrbST (n=74); and Tanestrous/TrbST (n=68).

Cows were artificially inseminated by the same technician with semen collected from Girolando bulls, which was obtained from the Brazilian Association of AI. Pregnancy was determined 30 days after AI by ultrasound evaluations and pregnancy rate was recorded in each treatment (number of pregnant cows divided by the total number of cows within the treatment).

### Statistical analysis

The data for follicle size, total number of follicles, diameter of the larger follicle, follicular growth rate, time between P4 implant removal and ovulation, and time from TAI and ovulation were evaluated by ANOVA and the treatment means compared by Tukey’s test considering effects of treatment, cyclicity, and the day of observation (8 and 10) using the PROC GLM [13]. Pregnancy rate was arranged in contingency tables and analyzed by Chi-square test (χ^2^). Differences among treatments were considered significant at α=0.05.

## Results

No interactions were observed between observation date and treatment, with the number of follicles and follicle classification (p>0.05; <6 mm, 6-8 mm, and >8 mm; Table-1).

**Table-1:** Number of follicles <6 mm, 6-8 mm, and >8 mm, on days 0, 8, and 10 according to experimental treatments and cyclicity (Average±SEM).

Item^1^	Number of follicles			

	<6 mm	6-8 mm	>8 mm	Total
Tratment^2^				
Tcontrol	10.1±0.6^a^	0.4±0.1^a^	0.9±0.1^a^	11.4±0.6^a^
TrbST	11.5±0.7^a^	0.6±0.1^a^	0.8±0.1^a^	12.9±0.7^a^
Cyclicity^2^				
Tcycling	10.4±0.7^a^	0.5±0.1^a^	0.9±0.1^a^	11.9±0.7^a^
Tanestrous	11.2±0.6^a^	0.4±0.1^a^	0.8±0.1^a^	12.4±0.6^a^
Day^3^				
0	9.8±1.0^a^	0.5±0.1^a^	0.4±0.1^b^	10.7±1.0^a^
8	11.3±0.7^a^	0.6±0.1^a^	1.1±0.1^a^	13.0±0.7^a^
10	11.3±0.7^a^	0.3±0.1^a^	1.1±0.1^a^	12.7±0.7^a^

1 Tcontrol=Control treatment, TrbST=Treatment 500 mg of rbST, Tcycling=Treatment with cycling cows, Tanestrous=Treatment with anestrous cows.

2,3 Means with different superscript letters in the same column differ by Tukey’s test (p<0.05). SEM=Standard error of mean

No effects were observed (p>0.05) for synchronization and cyclicity protocols, or the interaction of these factors with follicle growth rate, interval of removing the PRID, and TAI at ovulation (Table-2).

**Table-2:** FGR and removal interval of PRID and interval between the TAI and ovulation (average±SEM), according to synchronization protocols and cow’s cyclicity.

Item	FGR (mm/day)	Interval to ovulation	

		PRID removal (h)	TAI (h)
Treatment^1^			
Tcontrol	1.4±0.2^a^	64.6±1.4^a^	12.6±1.4^a^
TrbST	1.3±0.3^a^	64.4±1.4^a^	12.4±1.4^a^
Average	1.3±0.2^a^	64.5±1.0^a^	12.5±1.0^a^
Cyclicity^2^			
Tcycling	1.4±0.3^a^	64.9±1.3^a^	12.9±1.3^a^
Tanestrous	1.3±0.2^a^	64.0±1.6^a^	12.0±1.6^a^
Average	1.3±0.2^a^	64.5±1.0^a^	12.5±1.0^a^

1 Tcontrol=Control treatment, TrbST=Treatment 500 mg of rbST, Tcycling=Treatment with cycling cows, Tanestrous=Treatment with anestrous cows. FGR=Follicular growth rate, PRID=Progesterone-release intravaginal device, TAI=Timed artificial insemination, SEM=Standard error of mean

No interactions were observed (p>0.05) between treatment, cyclicity, and day of observation for the diameter and the diameter of the largest follicle (Table-3).

**Table-3:** Average diameter of the largest follicle according to treatment and cyclicity of cows on days 8 and 10 (average±SEM).

Diameter of the largest follicle (mm)					

Treatment^1^		Cyclicity^2^		Day^3^	
Tcontrol	TrbST	Tcycling	Tanestrous	8	10
11.0±0.6^a^	10.0±0.8^a^	10.9±0.6^a^	10.1±0.8^a^	9.2±0.6^b^	11.8±0.7^a^

1 Tcontrol=Control treatment, TrbST=Treatment with rbST, Means lacking a common superscript letter in a row differ by Tukey’s test (p<0.05).

2 Tcycling=Treatment with cycling cows, and Tanestrous=Treatment with anestrous cows,

3 Means lacking a common superscript letter in the same line differ by Tukey’s test (p<0.05). SEM=Standard error of mean

Reproductive efficiency according to treatments is shown in Table-4. Total pregnancy rate observed was 49.0% (n=150/306). Anestrous cows from TrbST had lower pregnancy rates (35.3%) compared to the cycling cows (56.8%; p<0.05).

**Table-4:** Pregnancy rate according to cyclicity and treatments.

Cyclicity/Treatment	TrbST	Tcontrol
Tcycling	56.8% (42/74)^aA^	51.2% (44/86)^aA^
Tanestrous	35.3% (24/68)^aB^	51.3% (40/78)^aA^

Similar lowercase letters within the same line do not differ (p>0.05). Different uppercase letters within the same column are significantly different (p<0.05).

## Discussion

The results found (Table-1) are in accordance with other authors, who reported a lack of rbST effects on these parameters in *B. t. taurus* and *B. t. taurus* **×** *B. t. indicus* dairy cows [14,15]. However, in the previous studies with *B. t. taurus* and *B. t. indicus*, the number of small follicles was increased by rbST [5,16,17]. As shown in Table-1, a lower number of follicles with a size >8 mm were observed on day 0 compared to days 8 and 10 (p<0.05). These results demonstrate that the improvement in physiological conditions, induced by the synchronization protocol using estradiol and progesterone in cycling and in anestrous cows, has led to an increase of the number of growing follicles as a result of the synchronization of the emergence of a new follicular wave. It can thus be inferred that the protocols used in this study, as well as those found in the literature, have satisfactory effects on controlling follicular and luteal dynamics and ovulation synchronization, allowing AI without previous estrus detection [18-20].

It has been reported that the administration of rbST increases the levels of IGF-I and consequently changes mainly the follicular growth due to a combination of direct effects of rbST and indirect effects of IGF-I [4]. The IGFs play an important role in follicular growth by stimulating the proliferation of granulosa cells in synergism with gonadotropins [6,7]. However, no effects of rbST on follicular growth were observed in this study.

Studies indicate that the administration of eCG in the protocol increases follicular growth rate since it improves the ovarian follicular development due to follicle-stimulating hormone and luteinizing hormone-like activity [21]. In this context, the present study suggests that all treatments were affected by eCG since the follicular growth rate observed is similar to those reported by other authors, who observed rates ranging from 1.3 to 1.8 mm/day in *B. t. taurus* using ovulation synchronization protocols [22,23]. Growth rates ranging from 0.7 to 1.8 mm/day in crossbred cows have also been found by other authors [24].

Treatment with rbST did not increase the size of the ovulatory follicle 48 h after the removal of the PRID (day 10) [14,15]. In a study with Holstein cows in Brazil, the ovulatory diameter was greater for cows treated with rbST (18.2 mm) compared to the control group (15 mm) [5].

The follicular diameter measured on day 10 (11.8 mm) was capable of inducing ovulation since 90.0% of the cows in the present study ovulated, independent of the treatment applied and their cyclicity. Therefore, there was no interaction between main effects and ovulation rate (p>0.05). The administration of rbST was thought to increase the ovulation rate due to the previously discussed factors such as the increase of plasmatic levels of IFG-I. However, ovulation rate was classified as excellent in all treatment groups minimizing the effects of rbST on improving the ovulation rate. Synchronization protocols of ovulation are well elucidated and efficient with regard to ovulation induction. Regardless of the inductors of ovulation used, the ovulation rates are >70% in the different categories (cows or heifers) and genetic groups (*B. t. taurus*, *B. t. indicus*, and *B. t. taurus **×** B. t. indicus*) [25,26].

The administration of rbST on the 1^st^ day of the protocol did not increase the pregnancy rate. Although the rbST dosage used in the present study was identical to that reported in studies that have shown positive effects on pregnancy rate, the lack of significant effects may have occurred due to the day of the treatment [9,27-29]. It is well known that with the administration of 500 mg of rbST, the plasmatic content of rbST and IGF-I remains elevated for 14 days [30]. In all cited studies, the rbST was administrated on the day of estrus, regardless if it was natural or synchronized by TAI protocols. However, in the present study, the rbST was administrated on the 1^st^ day of the TAI protocol, 10 days before estrus.

The rbST has been associated with a higher growth rate of the conceptus and consequently with an increase in pregnancy rate [9,28,29]. However, other studies report that the effect of rbST on the total pregnancy rate has not been observed [2,15]; or even a negative effect of rbST on reproduction, whereby cows treated with rbST had a decreased conception rate [31].

The results obtained in the present study are affected by several factors such as the dose of rbST and temperature, length of lactation, and mainly by the energy balance, which directly affects dairy cows’ reproduction and is not mentioned in most studies. In this context, factors that may affect the response to rbST administration must be considered when used as a tool to improve milk production and reproduction.

## Conclusion

The administration of rbST in a synchronizing ovulation protocol in crossbred cows did not affect the parameters of follicular dynamics studied here. A satisfactory pregnancy rate was observed; however, among the cows receiving rbST, anestrous cows had a lower pregnancy rate.

## Authors’ Contributions

FAO, JMP, and CAAT designed the concept for this research and scientific paper. All authors conducted the research. FAO, ICA, and LMS collected samples from the fields. All authors participated in the draft and revision of the manuscript and approved the final manuscript.
